# Implant-to-root dimensions projected by panoramic radiographs inthe maxillary canine-premolar region: implications for dental implant treatment

**DOI:** 10.1186/s12880-021-00567-7

**Published:** 2021-03-10

**Authors:** Annika Bertram, Alexander W. Eckert, Rüdiger Emshoff

**Affiliations:** 1 Otto Von Guericke University of Magdeburg, Magdeburg, Germany; 2 University Clinic of Oral and Maxillofacial Surgery, Martin-Luther University, Halle-Wittenberg, Germany; 3 University Clinic of Oral and Maxillofacial Surgery, Medical University of Innsbruck, Anichstraße 35, 6020 Innsbruck, Austria; 4 Private Practice, Oral and Maxillofacial Surgery, Freilassing, Germany

**Keywords:** Dental implants, Maxillary edentulousness, Panoramic radiography, Cone-beam computed tomography

## Abstract

**Backgound:**

This study aimed to compare panoramic radiography (PAN) and cone beam computed tomography (CBCT) determinations of implant-to-root dimensions (IRD) in anterior and posterior maxillary regions, and to help determine in which instances increased radiation exposure from CBCT scans may be justified.

**Methods:**

IRD measured by PAN (PAN-D) from implant-to-root sites (central incisor, lateral incisor, canine, first premolar, and second premolar) was collected from 418 implant sites in 110 adults. The CBCT technique was used as the reference method for the estimation of IRD. The PAN analysis equations were developed using stepwise multiple regression analysis and the Bland–Altman approach was applied to assess the agreement between PAN and CBCT methods.

**Results:**

The odds ratio that an implant at the canine-to-first premolar (9.7:1) (P = 0.000) or at the first premolar-to-second premolar region (4.5:1) (P = 0.000) belongs to the underestimation group was strong and highly significant. The root mean square error (RMSE) and pure error (PE) were highest for the canine-to-first premolar (RMSE = 0.886 mm, PE = 0.45 mm) and the first premolar-to-second premolar region (4.5:1) (RMSE = 0.944 mm, PE = 0.38 mm).

**Conclusions:**

This study provides evidence of site-specific underestimations of available horizontal bone dimensions for implants when assessed by PAN. These data suggest that the canines and first and second premolars may have to be excluded when assessing root angulations via PAN.

## Background

The success rates of implant surgery are reported to be as high as 95.1–97% [1–3]. However, surgical technique errors may occur with dental implant treatments such as abnormal implant angulations and implant malpositions [4, 5]. Furthermore, in specific regions where the implant is inserted, anatomical structures may be injured, including adjacent teeth roots, lingual and/or buccal bone plates, maxillary sinus membranes, the nasal cavity floor, and the mandibular canal [5, 6].

During implant placement, the alignment of the implant in an appropriate axial inclination to the neighboring teeth is critical for establishing and maintaining a correct and stable occlusal result. Especially in the narrow anatomical regions of the frontal maxilla, imaging techniques that display the exact localization and morphologic relation between the critical structures are required [7].

Conventional radiographs such as panoramic radiographs provide information regarding the vertical and mesio-distal relationships of implants with neighboring teeth and adjacent anatomical structures [7]. However, panoramic radiography (PAN) is affected by a certain degree of distortion in the horizontal and vertical planes. Several in vitro [8–10] and in vivo [11, 12] investigations have assessed angular distortion in PAN images, mainly addressing the aspect of tooth inclination. These studies have shown that PAN images are of limited use to evaluate mesiodistal angulations, and variations in root angulation are described to be greatest in the maxillary canine-premolar region [8–12]. In all of these published papers [8–12], it is not clear whether the PAN magnification factor given by the manufacturers were taken into consideration to calculate the respective measurement values. Furthermore, jaw site-specific magnification factors were not considered in any of these studies, i.e., the studies failed to take into account variations in jaw size and shape and errors in positioning the jaws in the machine [13, 14].

It may be questionable to base clinical decisions regarding implant insertion in the maxillary canine-premolar region on PAN findings, as the degree of distortion described for this region may result in an incorrect diagnosis and inappropriate treatment approach being applied to the patient. Compared to PAN techniques, cone-beam computed tomography (CBCT) imaging avoids the superimpositions of neighboring structures and the disadvantage of image magnification. In addition, CBCT presents a shorter scanning time and a radiation dose up to 15 times lower than that of multislice CT [15–18]. However, according to previous studies, there is little consensus regarding how much information CBCTs can provide over conventional radiographs and in which cases increased radiation exposure can be justified [19, 20].

The purpose of the present study was to compare panoramic and CBCT determinations of implant-to–root dimensions (IRD) in anterior and posterior maxillary regions and provide clinicians with practical guidelines to help determine in which instances adjunctive use of CBCT technology may be justified.

## Methods

### Study design

The subjects consisted of 110 consecutive adult patients (72 females and 38 males; average age 53.0 ± 15.2 years) referred to our practice of oral and maxillofacial surgery in Freilassing, Germany, for implant surgery. The subjects were informed about the study procedure, and verbal informed consent was received from each participant. Written informed consent was waived by the Institutional Ethics Committee as data were de-identified and analysed anonymously. This retrospective study followed the medical protocols and ethics outlined in the Declaration of Helsinki and was approved by the Medical Ethical Committee of the Martin-Luther University Institutional Review Board (ethics approval No. 2020-034). The inclusion criteria were age 18 years or older, partially or totally edentulous in the maxillary anterior or premolar region, and presence of post-implant complications or additional need for dental implants warranting concurrent panoramic and CBCT images taken after the postsurgical phase of implant surgery. The exclusion criteria for the study group included the presence of metallic artifacts that could impair an accurate analysis, distorted or unclear images (e.g., artifacts, scattering), pathology in the region of interest, and complete maxillary edentulism. The patients received 418 titanium Straumann® implants (Straumann AG, Basel, Switzerland) positioned in the central incisor, lateral incisor, canine, first premolar, and second premolar region of the maxilla. All patients underwent PAN and CBCT. The CBCT technique was used as the criterion method for the estimation of IRD.

### Imaging

Digital PAN was taken using the Orthophos SL 3D (ORT, Sirona Dental Systems GmbH, Germany), operating at 60–90 kVp and 3–16 mA. The magnification factor of the panoramic machine was 1.25. For CBCT imaging, the same Orthophos SL 3D machine was used. Images are rendered in a precise 1:1 ratio in the reconstruction software provided by the vendor. The scanning settings were as follows: 5 × 5.5 cm field of view, 85 kV tube voltage, 6–7 mA tube current, a radiation time of 14.1, and a 0.12 mm pixel size. By using the same protocol for all examinations performed, PAN and CBCT resulted in a radiation dose of 71 mGy cm^2^ and 218 mGy cm^2^, respectively. The effective dose from PAN and CBCT was 8.5 ySv and 26.2 ySv, respectively [21], i.e., the effective dose from a CBCT examination was about 3 times higher than that from the PAN examination. The radiographs were viewed with Galileos Implants and Sidexis 4.0 software (Dentsply Sirona).

### Measurement procedure

Implant-to-root sites (central incisor, lateral incisor, canine, first premolar, and second premolar sites) were assessed on each panoramic and CBCT radiograph. All radiographs were analyzed in standard conditions on a high-resolution grayscale SMM Series monitor (Siemens AG, Karlsruhe, Germany).

**Fig. 1:**
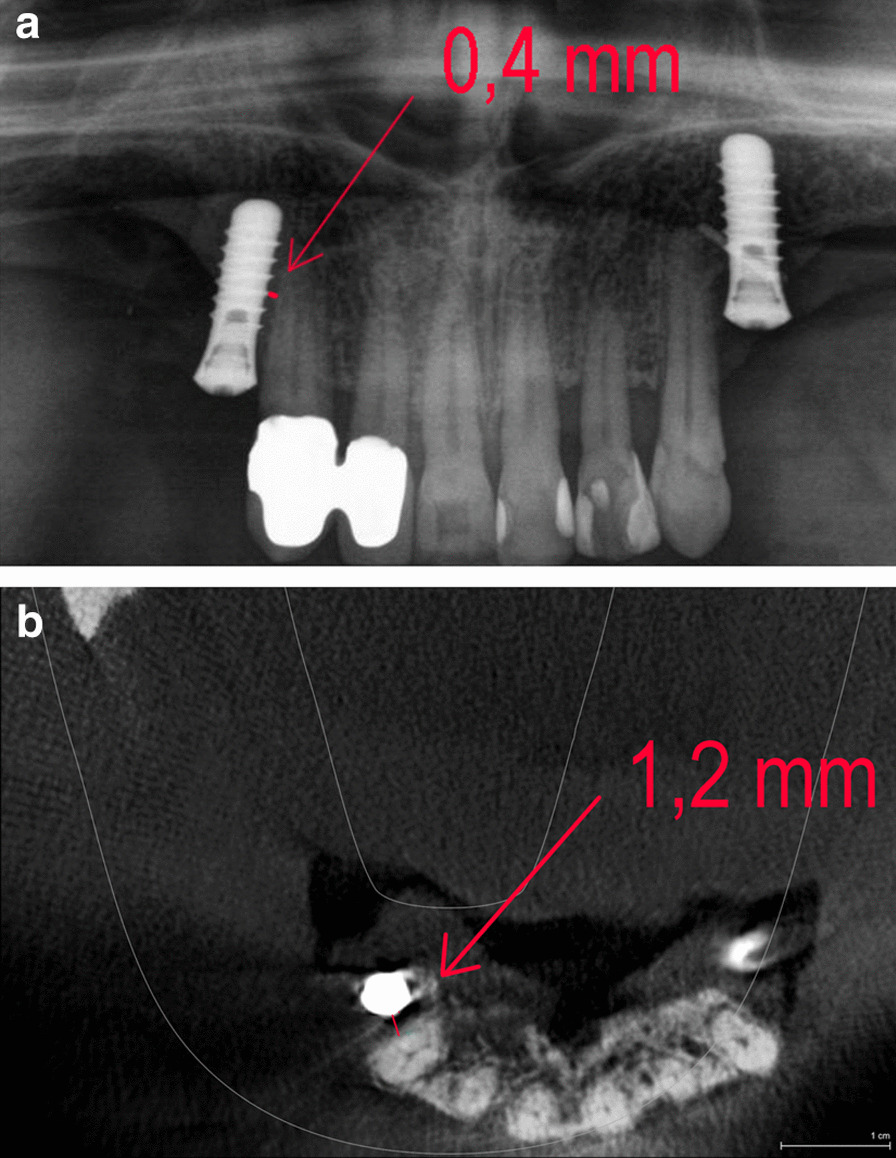
IRD measuring technique on PAN (**a**) and CBCT (**b**) without overlapping structures in PAN. **a** Outer surfaces of implant and neighboring root are assessed on PAN images. At the area of smallest distance between implant and adjacent root surface the local implant thread was used as reference point for IRD measurement (red line). **b** Outer surfaces of neighboring implant and root were assessed at the level of the reference thread on axial CBCT images. IRD represents distance between implant and adjacent root surface at the level of the reference thread (red line)

**Fig. 2:**
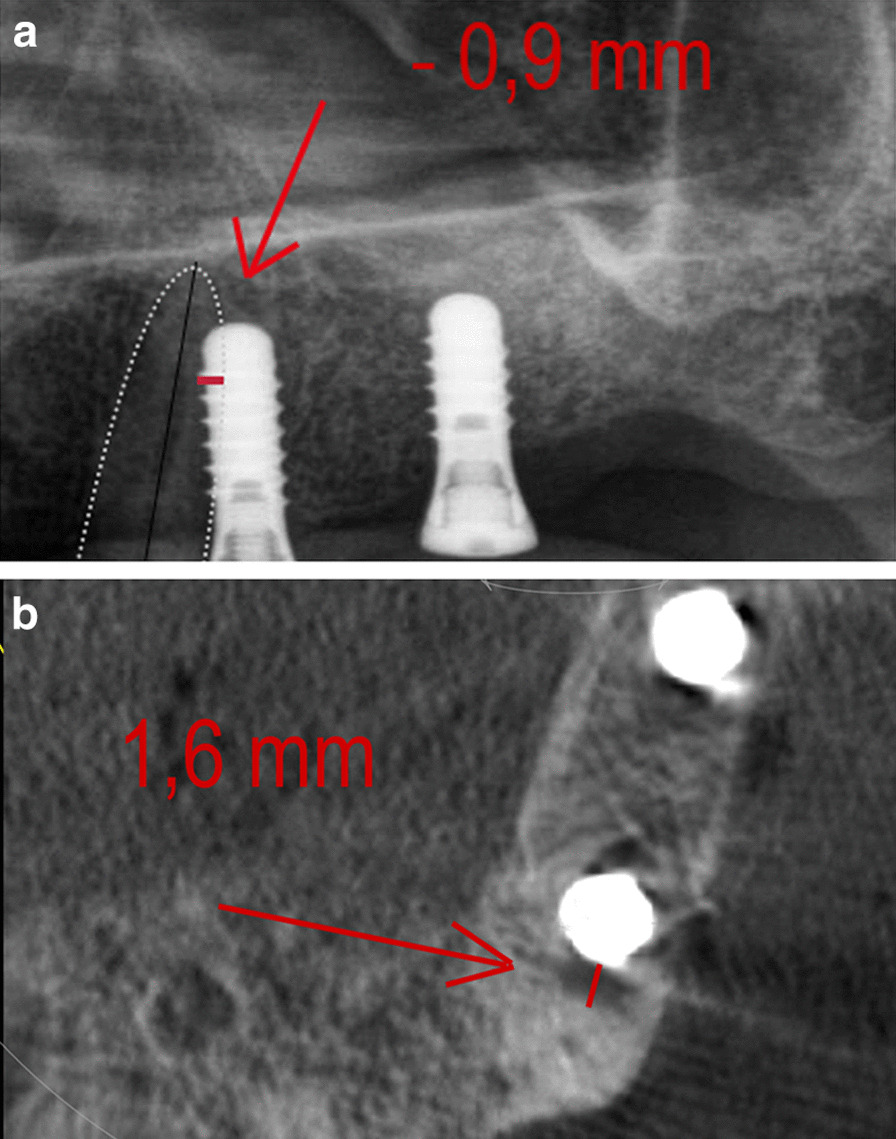
IRD measuring technique on PAN (**a**) and CBCT (**b**) with overlapping structures in PAN. **a** Outer surfaces of implant and neighboring root (dotted white line) are assessed on PAN images. At the area of smallest distance, i.e. greatest overlapping distance, between implant and adjacent root surface the local implant thread was used as reference point for IRD measurement (red line). **b** Outer surfaces of neighboring implant and root were assessed at the level of the reference thread on axial CBCT images. IRD represents distance between implant and adjacent root surface at the level of the reference thread (red line)

The shortest distance from the implant to the root of the neighbouring tooth was measured with PAN at sites corresponding to the central incisor, lateral incisor, canine, first premolar, and second premolar sites. The site-specifc multiplication factor was calculated for each implant site by dividing the implant’s measured length (in mm) on the postoperative PAN by the implant’s real length. The CBCT distances were measured on the correspondent axialbucco-palatal slices (Figs. 1 and 2). The measurements were made by a single examiner (AB) using a digital ruler.

For assessment of site-specific intraobserver reliability, IRD in the panoramic and CBCT images of 20 randomly selected cases were evaluated and measured by the investigator on two different days. For the panoramic and CBCT measurements, the mean differences were 0.27 ± 0.24 mm and 0.19 ± 0.16 mm, respectively, and the intraclass correlation coefficient for intraobserver agreement was 0.946 and 0.966, respectively (Table 1).

**Table 1:** Site-specific intraclass correlation coefficients of duplicate PAN and CBCT measurements of implant-to-root dimensions (n = 80)

Maxillary region	n	PAN			CBCT		
		M (mm)	SD (mm)	ICC	M (mm)	SD (mm)	ICC
Cen Inc–Lat Inc	20	0.11	0.12	0.989^#^	0.12	0.10	0.974^#^
Lat Inc–Canine	20	0.78	0.66	0.966^#^	0.14	0.09	0.931^#^
Canine–1. Prem	20	0.23	0.14	0.949^#^	0.17	0.15	0.952^#^
1. Prem–2. Prem	20	0.33	0.22	0.957^#^	0.27	0.22	0.960^#^
Total	80	0.27	0.24	0.946^#^	0.19	0.16	0.966^#^

*Cen* central, *Lat* lateral, *Inc* incisor, *Prem* premolar, *CBCT* cone-beam computed tomography, *PAN* panoramic radiography, *n* number of sites measured, *M* mean difference between the first and second measurements, *SD* standard deviation, *ICC* intraclass correlation coefficient, *mm* millimeters

^#^Accepted reliability

### Statistics

Apart from frequency, mean and standard deviation calculations, the statistical methods used were paired t-test and binary logistic and linear regression analyses. Binary logistic regression analysis was used for the assessment of the relative odds of each implant-to-root site. The outcome was always underestimation vs. nonunderestimation.

IRD derived from the CBCT method was used as the dependent variable for the development of prediction equations separately. The independent variables included IRD by PAN (Pan-D), age (to the nearest 1 year) and gender (male = 1, female = 0). The most predictive variables were selected by measure of goodness-of-fit statistics. A high R^2^ value, and small root mean square error (RMSE) indicated the optimal model. IRD assessed by the criterion method as well as the new prediction equations were compared using one-way analysis of variance (ANOVA). The pure error (PE), calculated as the mean of squares of differences between measured and predicted values, was used to assess the performance of the prediction equations. The smaller the PE is, the greater the accuracy of the equation [11].

Moreover, the approach of Bland and Altman was used to assess the agreement between the predicted and actual IRD. This statistical approach is recognized as the most appropriate way to compare the ability of different methods to measure the same parameter [12]. The 95% limits of agreement (expressed as minus and plus 1.96 standard deviations above and below the bias) were analyzed.

Significance was set at P < 0.05. For the statistical analysis, the NCSS 2019 statistical software (NCSS, LLC. Kaysville, Utah, USA) was used.

## Results

The distribution of the dental implants based on anatomic location is presented in Table 2. Eighty-five (20.3%) implants were inserted in the cental-lateral incisor region, 96 (23.0%) in the lateral incisor-canine region, 161 (39%) in the canine-first premolar region, and 76 (18%) in the first–second premolar region.

**Table 2:** Mean difference in implant-to-root dimensions by maxillary region as measured by PAN and CBCT (n = 418)

Maxillary region	n	PAN		CBCT		Difference		
		M (mm)	SD (mm)	M (mm)	SD (mm)	M (mm)	SD (mm)	P
Cen Inc–Lat Inc								
Cent Inc^I^–Lat Inc^T^	36	1.87	0.98	1.62	0.78	0.26	1.10	0.167
Lat Inc^I^–Cent Inc^T^	49	0.95	0.92	1.09	0.63	− 0.14	0.58	0.095
Total	85	1.34	1.04	1.31	0.74	0.25	0.85	0.759
Lat Inc–Canine								
Lat Inc^I^–Canine^T^	57	2.18	1.51	2.20	0.89	− 0.02	1.46	0.915
Canine ^I^–Lat Inc^T^	39	1.81	1.00	1.71	0.70	0.10	0.65	0.330
Total	96	2.03	1.33	2.00	0.85	0.03	1.19	0.810
Canine–1. Prem								
Canine ^I^–1. Prem^T^	41	0.30	1.12	1.54	1.00	− 1.24	1.47	0.000*
1. Prem ^I^–Canine^T^	120	0.87	1.10	1.93	1.01	− 1.06	0.91	0.000*
Total	161	0.73	1.13	1.83	1.02	− 1.11	1.08	0.000*
1. Prem–2. Prem								
1. Prem^I^–2. Prem^T^	41	1.10	1.40	1.63	1.31	− 0.54	0.98	0.000*
2. Prem^T^–1. Prem^I^	35	0.74	0.99	2.06	1.10	− 1.32	0.95	0.000*
Total	76	0.93	1.23	1.81	1.04	− 0.63	1.24	0.000*
Total	418	1.19	1.28	1.74	0.99	− 0.56	1.16	0.000*

*Cen* central, *Lat* lateral, *Inc* incisor, *Prem* premolar, ^*I*^ implant, ^*T*^ tooth, *CBCT* cone-beam computed tomography, *PAN* panoramic radiography, *n* number of sites measured, *M* mean, *SD* standard deviation, *mm* millimeters, *P* probability of type I error

*Significant difference with paired t-test

The mean implant-to-root dimension was 0.93 ± 1.23 mm on PAN and 1.74 ± 0.99 mm on CBCT. The difference in implant-to-root dimensions between the two radiographic techniques for the total material was − 0.56 mm ± 1.16 mm and ranged from − 1.32 mm (second premolar root-to-first premolar implant region) to 0.26 mm (central incisor-to-lateral incisor region). Statistically significant differences between the panoramic and CBCT techniques were found for the canine-to-first premolar (p = 0.000) and first premolar-to-second premolar region (p = 0.000) (Table 2).

The odds ratio that an implant at the canine-to-first premolar (9.7:1) (P = 0.000) and at the first premolar-to-second premolar region (4.5:1) (P = 0.000) belongs to the underestimation group was strong and highly significant. There was no significant increase in the odds ratio to indicate that an implant at the lateral incisor-to-canine region (0.8:1) (P = 0.493) would belong to the underestimation group (Table 3).

**Table 3:** Difference in implant-to-root dimensions by region as measured by PAN and CBCT (n = 418)

Diagnostic variables	Statistics				
	Estimate	Standard error	Odds ratio	95% CI	P
Underestimation^**#**^					
Lat Inc–Canine Region (n = 96)	− 2.09	0.305	0.81	0.45–1.48	0.493
Canine–1. Prem Region (n = 161)	2.30	0.339	9.95	5.12–19.31	0.000*
1. Prem–2. Prem Region (n = 76)	1.52	0.36	4.55	2.24–9.260.387	0.000*

*Cen* central, *Lat* lateral, *Inc* incisor, *Prem* premolar, *CBCT* cone-beam computed tomography, *PAN* panoramic radiography, *n* number of sites measured

#(PAN measurement − CBCT measurement) < 0, *P* probability of type I error

*Significant with logistic regression analysis adjusted for age and gender

A single regression equation was developed for the whole sample. Gender and age were not significant predictors of IRD (P > 0.05), with PAN-D (P = 0.000) entering the model and explaining the largest variance of the models. Figure 2 shows the relationship between CBCT-D and PAN-D (R^2^ = 25.6%; P = 0.000). Implant site-specific sets of preliminary equations were constructed for the prediction of CBCT-D. In each set, the equations were constructed using PAN-D as an independent variable. Implant site-specific PAN analysis prediction equations for CBCT-D were able to predict 25.0–43.4% of variances, while RMSE showed the highest values for the canine-to-first premolar (0.89 mm) and first premolar-to-second premolar region (0.94 mm) (Table 4).

**Table 4:** Equations for prediction of implant-to-root dimensions by region as measured by PAN and CBCT (n = 418)

Maxillary region	Equation	R^2^	RMSE (mm)
Cen Inc–Lat Inc (n = 85)	CBCT-D = 1.111 + −.008 Age + .440 PAN-D	.387	.586
Lat Inc–Canine (n = 96)	CBCT-D = .663 + .010 Age + .357 PAN-D	.434	.649
Canine–1. Prem (n = 161)	CBCT-D = 1.505 + .452 PAN-D	.250	.886
1. Prem–2. Prem (n = 76)	CBCT-D = 1.232 + .641 PAN-D	.415	.944

*Cen* central, *Lat* lateral, *Inc* incisor, *Prem* premolar, *CBCT-D* implant-to-root dimension by cone-beam computed tomography, *PAN-D* implant-to-root dimensions by panoramic radiography, *n* number of sites measured, *R*^*2*^ R square, *RMSE* root means-squared error

The developed regression equations were applied to the sample to evaluate their accuracy. The mean absolute difference between the predicted and measured CBCT values was 0.96 ± 0.86 mm. No significant difference between measured and predicted values for each tooth region was found (P > 0.05) (range of bias, − 0.20 mm to 0.14 mm). The highest PE was found for the canine-to-first premolar (0.45 mm) and first premolar-to-second premolar region (0.38 mm). The measured values strongly correlated with the the predicted values (range of r, 0.503 to 0.674, P < 0.0001) for IRD (Table 5).

**Table 5:** Implant-to-root dimensions assessed by criterion method and each of the PAN equations (n = 418)

Maxillary region	Mean (mm)	95% CI for the mean (mm)	ANOVA^a^ (P)	Correlation^b^ (r)	Mean bias (mm)	Pure error (mm)
Cen Inc–Lat Inc (n = 85)						
Criterion method (CBCT)	1.3 ± 0.7	1.16–1.47				
PAN equation	1.4 ± 0.4	1.27–1.46	0.564	0.568	− 0.05 ± 0.60	0.16
Lat Inc–Canine (n = 96)						
Criterion method (CBCT)	1.9 ± 0.8	1.73–2.07				
PAN equation	1.9 ± 0.6	1.82–2.06	0.744	0.612	− 0.03 ± 0.66	0.16
Canine–1. Prem (n = 161)						
Criterion method (CBCT)	1.8 ± 1.0	1.67–1.99				
PAN equation	1.7 ± .05	1.61–1.77	0.109	0.503	0.14 ± 0.88	0.45
1. Prem–2. Prem (n = 76)						
Criterion method (CBCT)	1.8 ± 1.2	1.55–2.11				
PAN equation	2.0 ± 0.6	1.90–2.15	0.207	0.674	− 0.20 ± 0.95	0.38

*CBCT* cone-beam computed tomography, *PAN* panoramic radiography, *Cen* central, *Lat* lateral, *Inc* incisor, *Prem* premolar, *ANOVA* analysis of variance, *P* probability of type I error

^a^Comparison of means between criterion method and assessments made by each of the prediction equations, *r* regression coefficient

^b^Correlation between criterion method and assessments made by each of the prediction equations

**Fig. 3:**
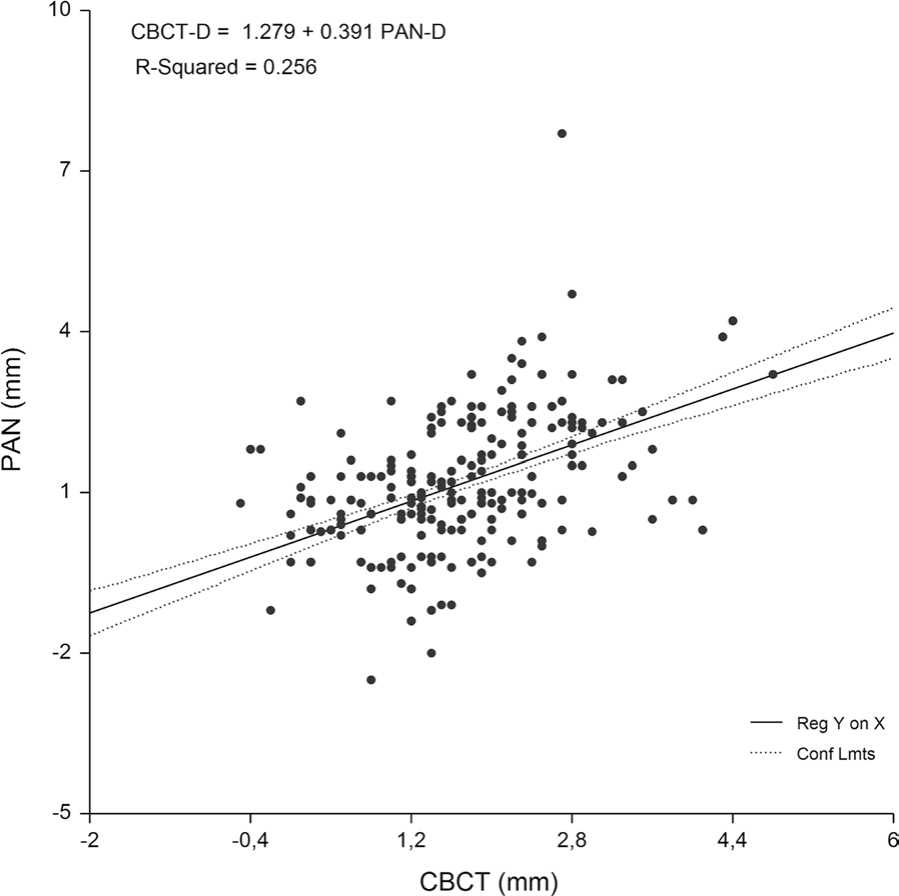
Linear regression of the relationship between IRD assessed by PAN and criterion method (CBCT)

**Fig. 4:**
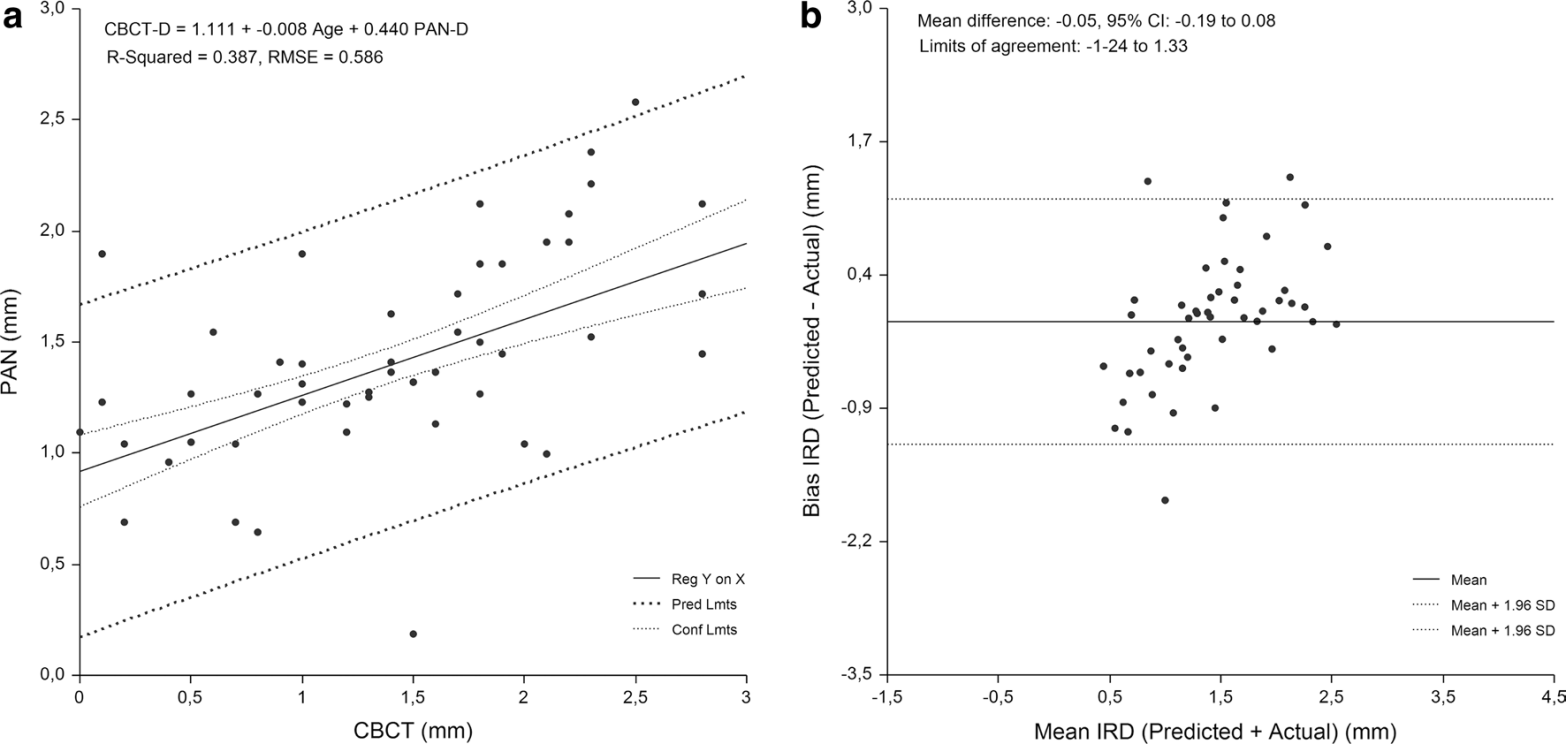
Central incisor-to-lateral incisor region. Linear regression (**a**) and Bland Altman analysis (**b**) of the relationship beween IRD assessed by PAN analysis prediction equation and criterion (CBCT) method. RMSE = 0.586 mm, mean bias = -0.05 mm and PE = 0.16 mm

**Fig. 5:**
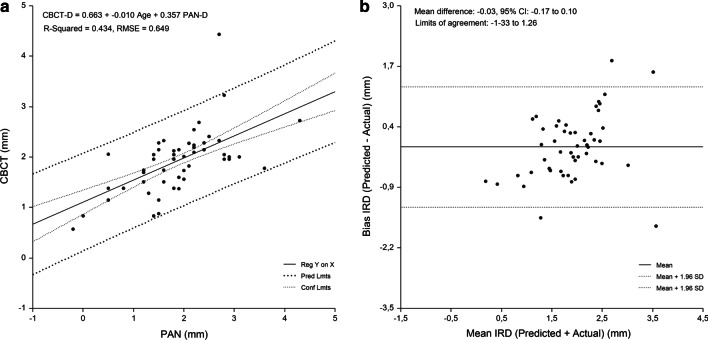
Lateral incisor-to-canine region. Linear regression (**a**) and Bland Altman analysis (**b**) of the relationship beween IRD assessed by PAN analysis prediction equation and criterion (CBCT) method. RMSE = 0.649 mm, mean bias = -0.03 mm and and PE = 0.16 mm

**Fig. 6:**
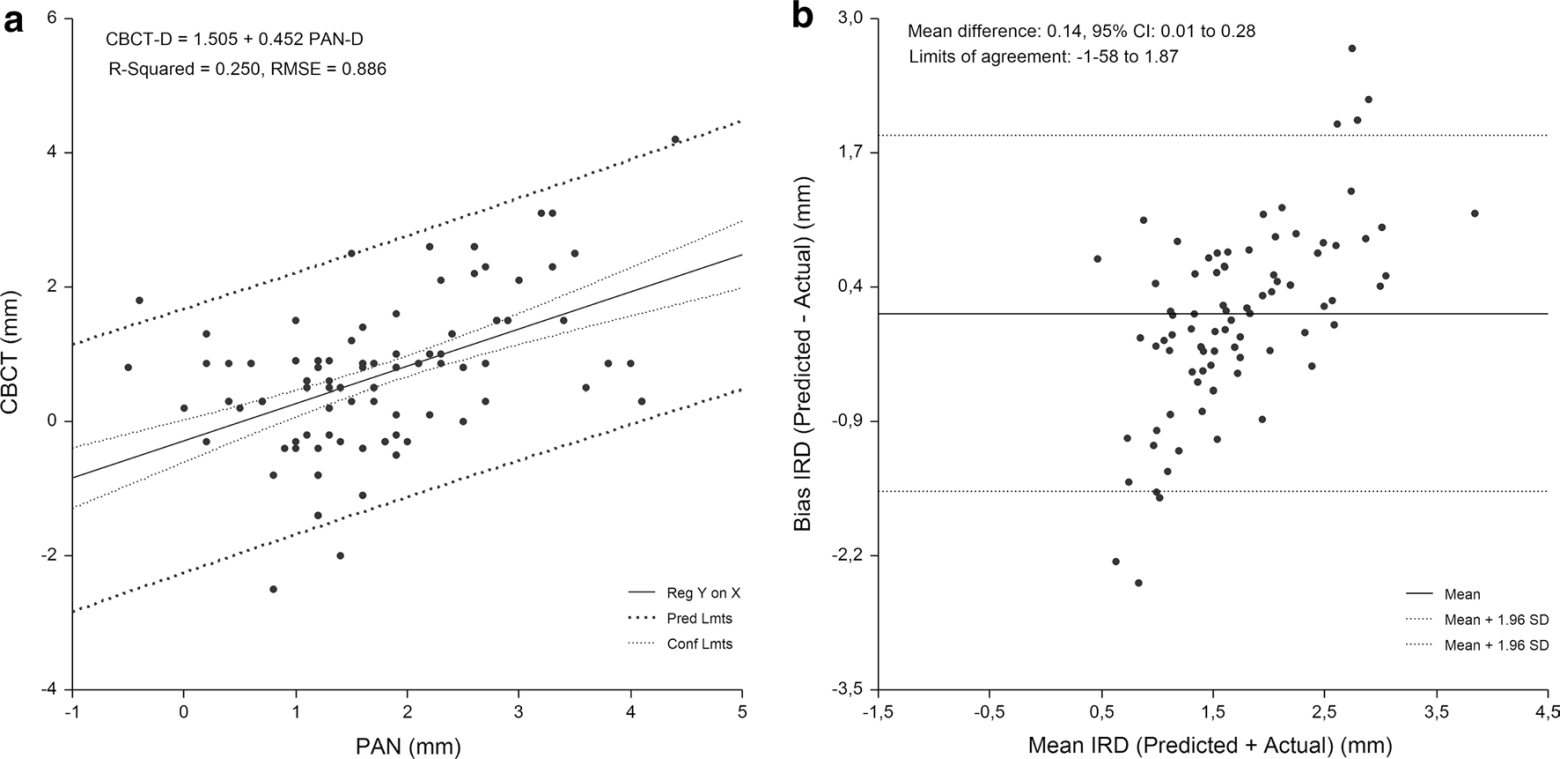
Canine-to-first premolar region. Linear regression (**a**) and Bland Altman analysis (**b**) of the relationship beween IRD assessed by PAN analysis prediction equation and criterion (CBCT) method. RMSE = 0.886 mm, mean bias = 0.14 mm and PE = 0.45 mm

**Fig. 7:**
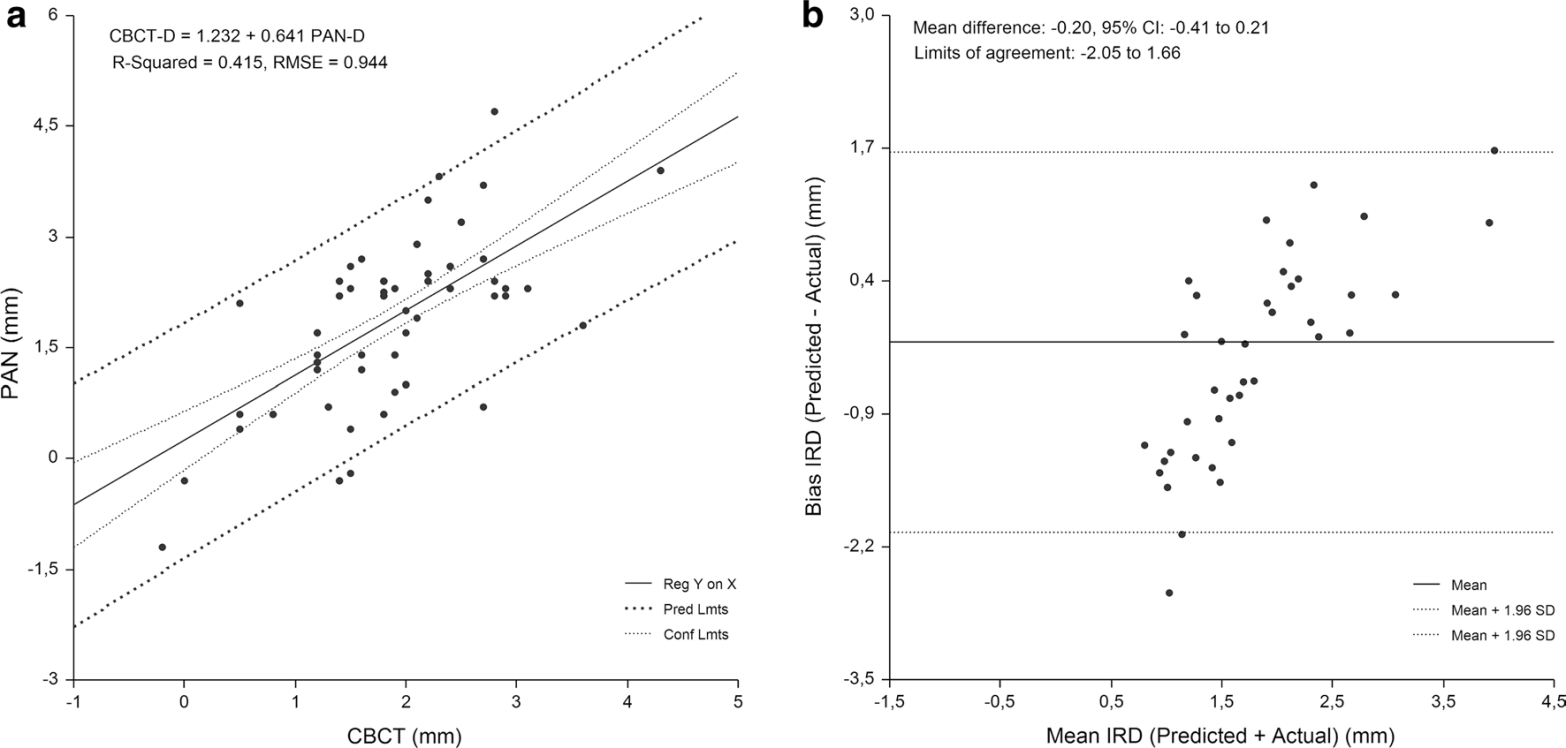
First premolar-to-second premolar region. Linear regression (**a**) and Bland Altman analysis (**b**) of the relationship beween IRD assessed by PAN analysis prediction equation and criterion (CBCT) method. RMSE = 0.944 mm, mean bias = -0.20 mm, and PE = 0.38 mm

The linear relationship between the measured and predicted IRD and the difference between the measured and predicted IRD plotted against the mean of the predicted and measured IRD are shown in Figs. 3, 4, 5, 6 and 7. Bland–Altman analyses showed the lowest agreement between predicted and actual IRD for the canine-to-first premolar (limits of agreement, − 1.58 mm to 1.87 mm) and first premolar-to-second premolar region (limits of agreement, − 2.05 mm to 1.66 mm). A total of 19 implant-sites (4.6%) with residuals exceeding the 95% confidence limits of IRD, were identified.

## Discussion

The mean difference in IRDs between the two radiographic techniques was − 0.56 ± 1.16 mm. The differences between the two radiographic techniques ranged widely for IRDs. The highest negative values were found for canine-to-first premolar (− 1.11 ± 1.08 mm) and first-to-second premolar regions (− 0.63 ± 1.24 mm), i.e., PAN underestimates available horizontal bone dimensions in these regions. These findings seem to be similar to those of Tepedino et al. [22], who reported that calibrated PAN overall underestimates the available interradicular space in comparison to CBCT. However, they may contradict the reports of Bouwens et al. [11] and Peck et al. [12], who described that in comparison to CBCT, uncalibrated PAN projects the largest diversion of root angulation in the maxillary canine [11] and maxillary canine-premolar regions [12]. The findings in the latter studies [11, 12] could be contradictory because these authors did not consider the impact of jaw site-specific magnification factors, i.e., these studies failed to take into account the aspect of jaw size and shape variations and the occurrence of jaw positioning errors.

It is of clinical importance to understand and anticipate PAN-associated deviations in axial tooth positions. Significant inaccuracies in mesiodistal tooth angulations found in PAN were described by previous investigators. PAN inaccuracies have been reported to include variable vertical and horizontal magnification factors, projection geometry, focal trough depth and geometry, and positioning errors of the patient [23–26].

Several authors showed significant differences with regard to the localization of apices in the mesio-distal directions following 2D examination [27, 28]. They described a greater tendency in the first premolar region, caused by horizontal distortions on PAN images that occur in instances where the object image is located in front or behind of the focal trough [7, 29]. Furthermore, different face shapes may lead to varying maxillary dentition positionings within the focal trough, thereby causing aberrant radiographic angulations. Further investigations may be warranted to address this important issue [8].

Clinicians frequently use PAN before and during dental implant treatment to assess mesiodistal tooth angulations. The appearance of a change in mesiodistal tooth angulation may be due to varying inclinations of inserted implants and neighboring teeth in the buccolingual direction. It has been shown that an increased lingual root torque may appear as a more mesial root tip on the PAN, while an increased buccal root torque may result in a more distal root tip. Inconsistency and extensive variability have been reported regarding the effect of buccolingual angulation on mesiodistal angulation [30, 31].

PAN underestimated the available interradicular spaces in the canine-to-first premolar and first-to-second premolar regions compared with CBCT, which is considered the gold standard for linear measurements [32]. These results could be explained by the fact that the arch displays an increased curvature at the canine region, and PAN images therefore present greater distortion [10]. Furthermore, the first premolar usually has two roots and is located in a more anterior zone where the alveolar ridge is usually thinner, whereas the second premolar usually has just one root and is located in a more posterior zone where the alveolar ridge tends to widen.

It is important to insert dental implants in a functionally and esthetically correct position, i.e., dental implants must be placed in correct positions and angulations in relation to each other and to adjacent teeth. The present study provides a perspective on the contribution of tooth region variables of panoramic IRD dimensions to the occurrence of underestimation of available horizontal bone dimensions. While the lateral incisor-to-canine region contributed no amounts to the change in risk (1:0.81), a clear definition of the underestiamtion group was evident for the canine-to-first premolar (1: 9.95) and first-to-second premolar regions (1: 4.55). Therefore, based on this study, tooth region may be considered a dominant factor in the underestimation of available bone dimensions. Further investigations are indicated to clarify which additional variables may be associated with an elevated risk of underestimation of available horizontal bone dimensions.

The current study developed PAN analysis prediction equations for the estimation of IRD in the anterior and posterior maxilla. To the best of our knowledge, this is the first study to develop a PAN equation for adults across tooth region-specific groups. The developed PAN equations showed comparatively minor predictive performance for the canine-to-first premolar (R^2^ = 0.25%, RMSE = 0.89 mm) and the first-to-second premolar region (R^2^ = 0.42%; RMSE = 0.94 mm). Furthermore, the validation results indicated that PE and bias were comparatively higher for these regions, while the limits of agreement assessed by the Bland–Altman approach showed comparatively wider ranges. These data support recent changes by the American Board of Orthodontics to exclude scoring of the canines when assessing root angulations via PAN [33]. However, the data may also indicate that it would be judicious to exclude first and second premolars evaluation by PAN. Furthermore, these findings may suggest that the equations may not be suitable for use in a community or clinical setting when CBCT techniques are not available. This suggests that caution should be applied when interpreting canine, premolar, and molar angulation. Use of CBCT may therefore be recommended for all implant size and angulation estimations in these regions.

In implant dentistry, CBCT imaging has been considered a highly accurate treatment planning tool for the performance of reliable linear measurements [15, 34]. However, there are several factors, such as machine characteristics, radiation exposure, and image-processing software, that may affect the accuracy of reformatted CBCT images [35, 36]. In a recent systematic review of the available evidence on the accuracy of linear measurements when using maxillofacial CBCT specifically in the field of implant dentistry [37], the authors reported that most studies showed submillimeter accuracy of CBCT measurements compared to a gold standard, and there was no clear trend as to whether measurements are consistently under- or overestimated.

The present study needs to be evaluated in the context of some limitations. First, measurements were made by a single observer, i.e., observer bias could have occurred in the data collection process. This error may be reduced by study designs incorporating two or more observers and a multicenter setting with comparison and statistical correlation of their measurements. Second, the use of CBCT imaging can produce artifacts caused by high-density metal materials such as dental implants [38, 39], while CBCT has limited capability to decrease artifacts with use of metal artifact reduction algorithms or correct exposure settings [40, 41]. Although these artifacts occur more frequently in the mandible than in the maxilla, and are generated most prominently by zirconium implants, followed by titanium zirconium and titanium implants [42, 43], beam hardening and scattering effect artifacts could have reduced the contrast, thereby impairing the detection of structures of interest and as a result producing errors in the linear measurements on CBCT images.

## Conclusions

This study provides evidence of site-specific underestimations of available horizontal bone dimensions for implants when assessed by PAN. These data may suggest that the canines andfirst and second premolars should be excluded from assessments of root angulations via PAN. Use of CBCT may therefore be recommended for all implant size and may have to be excluded from assessments of angulation estimations in these regions.

## Data Availability

Due to the nature of this research, participants of this study did not agree for their data to be shared publicly, so supporting data is not available.

## Abbreviations

ANOVA: One-way analysis of variance
CBCT: Cone-beam computed tomography
CBCT-D: IRD assessed by CBCT
IRD: Implant-to-root dimensions
PAN: Panoramic radiography
PAN-D: IRD assessed by PAN
RMSE: Root mean square error
PE: Pure error
